# Parent Language Input Prior to School Forecasts Change in Children’s Language-Related Cortical Structures During Mid-Adolescence

**DOI:** 10.3389/fnhum.2021.650152

**Published:** 2021-08-02

**Authors:** Ö. Ece Demir-Lira, Salomi S. Asaridou, Collin Nolte, Steven L. Small, Susan Goldin-Meadow

**Affiliations:** ^1^Department of Psychological and Brain Sciences, University of Iowa, Iowa City, IA, United States; ^2^DeLTA Center, University of Iowa, Iowa City, IA, United States; ^3^Iowa Neuroscience Institute, University of Iowa, Iowa City, IA, United States; ^4^Department of Experimental Psychology, University of Oxford, Oxford, United Kingdom; ^5^Department of Biostatistics, University of Iowa, Iowa City, IA, United States; ^6^School of Behavioral and Brain Sciences, University of Texas at Dallas, Richardson, TX, United States; ^7^Department of Psychology, University of Chicago, Chicago, IL, United States

**Keywords:** MRI, language acquisition, brain structure, parental language input, language development, cortical thickness

## Abstract

Children differ widely in their early language development, and this variability has important implications for later life outcomes. Parent language input is a strong experiential factor predicting the variability in children’s early language skills. However, little is known about the brain or cognitive mechanisms that underlie the relationship. In addressing this gap, we used longitudinal data spanning 15 years to examine the role of early parental language input that children receive during preschool years in the development of brain structures that support language processing during school years. Using naturalistic parent–child interactions, we measured parental language input (amount and complexity) to children between the ages of 18 and 42 months (*n* = 23). We then assessed longitudinal *changes* in children’s cortical thickness measured at five time points between 9 and 16 years of age. We focused on specific regions of interest (ROIs) that have been shown to play a role in language processing. Our results support the view that, even after accounting for important covariates such as parental intelligence quotient (IQ) and education, the amount and complexity of language input to a young child prior to school forecasts the rate of change in cortical thickness during the 7-year period from 5½ to 12½ years later. Examining the proximal correlates of change in brain and cognitive differences has the potential to inform targets for effective prevention and intervention strategies.

## Introduction

Language skills are fundamental for children’s later life outcomes (e.g., Duncan et al., 2007; Marchman and Fernald, 2008; Bleses et al., 2016). Variability in children’s language skills early in life has been linked to variability in children’s home environments. Indeed, one of the best-established findings in the developmental literature is that variability in children’s early language skill is influenced by the quantity and quality of language input they receive from their parents (e.g., Huttenlocher et al., 1991, 2002; Hart and Risley, 1995; Weizman and Snow, 2001; Hoff, 2003; Rowe and Goldin-Meadow, 2009; Rowe et al., 2009; Cartmill et al., 2013). Variability in early child language skills have also been shown to predict variability in later structural brain differences in language areas. For example, vocabulary growth measured at age 14–58 months predicts cortical thickness in the left supramarginal gyrus (SMG) at age 8 to 10 years old (Asaridou et al., 2017). However, less is known about the relation between children’s experiential factors early in life and *change* in later brain structures. Here, we attempt to bridge this gap using a unique longitudinal data set spanning 15 years. We ask whether parental language input during preschool years predicts *changes* in later (mid-adolescent) cortical structures that subserve language processing, over and above possible covariates such as parental socioeconomic status (SES) or intelligence quotient (IQ).

## Parental Language Input and Child Language Development

Previous behavioral work highlights the role of parental cognitive stimulation, and the role of parental language input more specifically, in shaping children’s cognitive outcomes. One of the most frequently reported findings in the developmental literature is the association between early parental language input and language development (e.g., Huttenlocher et al., 1991, 2002; Hart and Risley, 1995; Weizman and Snow, 2001; Hoff, 2003; Rowe and Goldin-Meadow, 2009; Rowe et al., 2009). Language input more strongly predicts child language outcomes than SES or a variety of other characteristics of parent–child interactions, such as parental affect. Measures of language input often focus on its quantity, such as the number of word tokens parents produce (Huttenlocher et al., 1991; Hart and Risley, 1995; Weizman and Snow, 2001; Rowe, 2008, 2012; Barnes, 2011; Weisleder and Fernald, 2013; Demir-Lira et al., 2019; Rowe and Snow, 2020). More recent research has also highlighted the complexity of language input, such as parental use of rare words or talk about abstract topics, as a predictor (e.g., Demir et al., 2015; Rowe and Snow, 2020; see also Cartmill et al., 2013). In the current paper, to gain a comprehensive view of children’s input, we consider both the quantity and complexity of early parental input in predicting later child brain structure.

## Brain Areas Associated With Language Development

A wide set of networks in the brain supports language development. One network, particularly specialized for language, includes (among other regions) the superior temporal gyrus (STG), superior temporal sulcus (STS), middle temporal gyrus (MTG), SMG, and inferior frontal gyrus (IFG; pars opercularis and pars triangularis) (Wilke et al., 2009; Friederici and Gierhan, 2013). Among other roles, STG is thought to be involved in speech perception (Hickok and Poeppel, 2007), MTG in semantic processing (Price, 2012), the SMG in phonological processing (Rodríguez-Fornells et al., 2009), and the IFG in speech processing and lexical competition (Davis and Gaskell, 2009; Rodríguez-Fornells et al., 2009; Price, 2010, 2012; Fedorenko and Thompson-Schill, 2014; Li et al., 2014). Brain structure in these regions of interest (ROIs) is related to children’s language skills. For example, left IFG, MTG and STG volumes differentiate typically-developing children from children with language disorders (e.g., Badcock et al., 2012; Lee et al., 2020).

The focus of the current paper is on brain structure, specifically, cortical thickness, because underlying cellular components of cortical thickness are amenable to *change* as a result of postnatal experience and learning (Diamond et al., 1964; Black et al., 1990; Anderson et al., 1994; Kleim et al., 1996). Cortical thickness is measured by the distance between the boundary of white and cortical gray matter, and gray matter and the pia mater. Cortical thickness varies roughly between 2 and 4 mm, with frontal and occipital poles being thinnest and temporal and insular cortices being thickest (Ribeiro et al., 2013). Although, as a general trend, cortical thickness decreases over childhood and early adolescence, ultimately plateauing in early- to mid-adulthood, development varies across cortical regions. Some regions, such as temporal areas, exhibit less linear and more quadratic patterns of development than other areas (Sowell et al., 2003; Raznahan et al., 2011; Mutlu et al., 2013; Mills et al., 2016).

## Parental Language Input and Brain Areas Associated With Language Development

Discussions of the role that parental input plays in language development rarely include the underlying neural basis of this development. When experiential factors have been considered in relation to the neurobiological basis of language processing, parental SES (typically measured by family income, parental educational attainment, and/or parental occupational prestige) has been the focus (Duncan and Magnuson, 2012). For example, SES disadvantage has been associated with reduced volume (e.g., Jednoróg et al., 2012; Hair et al., 2015), thickness (Mackey et al., 2015), and surface area (Noble et al., 2015) in cortical regions underlying language comprehension, including perisylvian areas (e.g., STG) and ventrolateral prefrontal areas (e.g., IFG; Noble et al., 2012; Piccolo et al., 2016). SES-related differences are also observed in white matter structures, and in functional brain systems, involved in language processing (Raizada et al., 2008; Gullick et al., 2016; Younger et al., 2019). However, parental SES is a complex construct of many components (e.g., parental income, education, and neighborhood characteristics). Any one of these components of SES could be influencing children’s academic outcomes via more day-to-day interactions, such as parental language input.

A few recent studies have begun to examine the associations between parental language input and brain structure and function for language processing. Avants et al. (2015) found associations between HOME, an observational measure of the home environment, and later cortical thickness in areas central to language processing. Using naturalistic recordings of parent–child conversations in the home, Romeo et al. (2018a) showed that, in 4 to 6-year-old children, the number of conversational turns with adults in the home environment (a measure of input complexity), predicts left IFG activation during a story-listening task completed at the same age, and that number of turns mediates the relation between SES and children’s language skill, as well as white matter connectivity in left arcuate and superior longitudinal fasciculi, also at the same age (Romeo et al., 2018b). Building on this work, Merz et al. (2020) found that the greater the input quantity (number of adult words) and complexity (number of conversational turns) in 5- to 9-year-old children, the greater left perisylvian cortical surface area in these children at the same age. The input quantity and complexity measures were highly correlated and revealed similar associations.

We add to this small but growing literature in several ways. First, previous investigations examined *concurrent* relations between parental input and child brain structure. However, to explore predictive relations, we need to examine parental input early in development, and child brain structure later in development in the same children – the focus of this paper. Second, previous studies measured brain structure at a single time point, but parental input might have different effects on child measures if those measures are taken longitudinally (e.g., Rowe et al., 2012, with respect to behavioral measures; Piccolo et al., 2016, with respect to brain measures). Here, we assess *changes* in brain structure over time during development. Third, existing studies rely on recording devices (e.g., LENA devices) that provide automatized measures, but do not produce transcription of audio recordings. Past studies leveraged conversational turns as a measure of input complexity, which is automatically calculated by LENA. However, conversational turns do not reveal the specific linguistic features that are predicting later outcomes. Here, we consider measures not only of input quantity, but also of input complexity, which requires hand-coding. Fourth, the youngest children included in previous studies of parent language input–child brain structure relations were 4 years old; however, by 4, children already vary greatly in their language skills (Fenson et al., 1994). Recent work shows that early parental input may predict later child outcomes better than input in later preschool years (Rowe et al., 2012). Here, we focus on parental input beginning at child age 18 months. Finally, we examine the relation of this early parental input to child brain structure in mid-adolescence, a much later age than has typically been studied.

## Current Study

Ours is the first study to examine predictive, longitudinal relations between early parental language input and *changes* in child brain structure over time. We examine the relation between two measures of early parental input – quantity and complexity – between child age 18 and 42 months, and changes in child cortical thickness between 9 and 16 years of age. To do so, we gathered a range of input measures collected directly from naturalistic interactions in the home at child age 18–42 months, when children already show great variability in language development. We then assessed children’s brain structure at five different time points between 9 and 16 years of age. We focus on brain regions that have been shown to play a particularly strong role in language processing. We also focus on cortical thickness as our measures of brain structure. Cortical thickness is tied to the number of neurons in a cortical column, the amount of glial and capillary support, and dendritic branching (Rakic, 1988, 2009), all of which are amenable to change as a result of postnatal experience and learning and thus deem cortical thickness as particularly sensitive to environmental experiences (Black et al., 1990; Anderson et al., 1994; Kleim et al., 1996). Our main research question is how parental language input during preschool years relates to changes during mid-adolescence in child brain structures involved in language processing. Based on prior behavioral and neuroimaging literature, we hypothesize that parental language input will positively predict both average cortical thickness and *changes* in cortical thickness, controlling for parent background variables, such as parent income, education and IQ.

## Materials and Methods

### Participants

Twenty-three children (12 female) participated in the study. All were native speakers of American English and were studied over a 15-year period. The children were drawn from a sample of 64 children participating in a larger, longitudinal study of children’s language development in the greater Chicago area (see Goldin-Meadow et al., 2014). Participants were recruited from the Chicago area via mailings to families in targeted zip codes and via an advertisement in a free parent magazine. A subset of the 64 children from the original sample agreed to participate in the neuroimaging component of the larger study (*n* = 23); these are the families described in this study. Each parent gave written informed consent following the guidelines of the Institutional Review Boards for the Division of Biological Sciences at The University of Chicago, and the Office of Research at the University of California, Irvine, which approved the study. Children gave verbal assent. All participants reported normal hearing and normal or corrected-to-normal vision. No parent reported any history of neurological or developmental disorders in their child. Handedness was assessed using the Edinburgh handedness inventory (Oldfield, 1971).

Parent language input measures were collected at the 18, 30, and 42 month behavioral visits (see procedure below). A total of 30 participants were tested in the Magnetic resonance imaging (MRI) component over the 5 years between 9 and 16 years of age. Seven participants were excluded from the analyses because they did not have the early parental input data, resulting in a final sample of 23 families. As described below, children were scanned a maximum of five times – a number of individual MRI sessions were excluded because the child failed to complete the session or moved excessively (more than 10% of the total number of volumes).

According to parent report, 19 children were White, 2 were African–American, and 2 were of mixed race. In terms of ethnicity, 3 of the children were reported to be Hispanic and 20 were non-Hispanic. Parent education (in years) was coded on a categorical scale (10 = less than high school degree, 12 = high school degree, 14 = some college or associate degree, 16 = college degree, 18 = more than college). In this sample of 23 children, average parent education was 15.6 years (SD = 2.4, range = 10–18) and average family income was $59,456 (SD = $30,738, range = $7500–$100,000). For 22 children, mother was the primary caregiver; for 1 child, father was the primary caregiver. All but 3 of the families reported the education level of a secondary caregiver as well. For these 20 families, education levels for the primary and secondary caregivers were highly correlated, *r* = 0.57, *p* = 0.008. Because family income and caregiver education were highly correlated, the two were combined using a principal components analysis (PCA), which returned a single composite measure for SES. Correlation between SES composite and education is ρ = 0.86, and correlation between SES composite and income is ρ = 0.69. SES captured 47% of the variability between education and income.

### Behavioral Procedure

The parental language input included in this study was collected as part of the larger longitudinal study described previously (see Goldin-Meadow et al., 2014). We coded videotapes of parents interacting with their children for approximately 90 min during home visits that occurred every 4 months between child ages 14–58 months. Parents were not given any specific instructions and were asked to engage in their normal daily activities. Typical activities included toy play, book reading, and eating meals and snacks. In the current study, three visits were chosen (visits at child age 18, 30, and 42 months). We focused on these three time points for multiple reasons: (1) previous research using data from the larger sample showed significant relations between input provided at these three time points and later child outcomes, highlighting the role of children’s early experiences (Rowe et al., 2012); (2) the earlier the ages, the lower the possibility of children directing the input parents provide to them, (3) recent work has shown that parents tend to be stable in their input in these early years (Silvey et al., 2021); and finally, (4) input in earlier preschool years, compared to the entire preschool period up to 58 months, reveals similar relations to later outcomes (Rowe et al., 2012).

### Behavioral Measures

#### Parent Language Input Measures

All parent and child speech in the videotaped sessions were transcribed. Only speech directed to the child was used in the current analyses based on previous work suggesting that language directed to the child might be more strongly related to child language development than overheard speech (Shneidman et al., 2013; Weisleder and Fernald, 2013). The unit of transcription was the utterance. An utterance was defined as any sequence of words that was preceded and followed by a pause, a change in conversational turn, or a change in intonational pattern. Transcription reliability was established by having a second individual transcribe 20% of the videotapes with a reliability criterion of 95% agreement on utterance transcription. Our measures of input consisted of three different components: (1) the number of word tokens, (2) number of rare words, and (3) decontextualized utterances parents produced at child age 18, 30 and 42 months during the 90-min visits. Word tokens were the total number of words parents produced. Rare words were identified using the method described by Beals and Tabors (1995) (see also Weizman and Snow, 2001). We removed all non-dictionary words from the corpus of spoken parent words and the most common words (and all their inflected forms) known by fourth graders, as judged by teachers, and compiled in the Dale-Chall word list (Dale and Chall, 1948; Chall and Dale, 1995). The remaining words in the parent input corpus were considered rare words. Decontextualized language utterances produced by parents and children were identified and coded as in Rowe (2012). Categories of decontextualized language included narrative, pretend, and explanation (see Rowe, 2012; Demir et al., 2015; for detailed definitions of each category). All utterances marked as narrative, pretend or explanation were considered decontextualized. Since effectiveness of specific input features varies by child age, we focused on features of the input that have been shown to predict child language outcomes during the period observed (Rowe and Snow, 2020; Silvey et al., 2021). We also excluded interactional aspects of the input, such as conversational turns, that might reflect broader characteristics of the parent–child interactions, such as parent sensitivity or parent–child synchrony.

#### Parent IQ

Parent verbal IQ was measured using Wechsler Abbreviated Scale of Intelligence (WASI-II, Wechsler, 2011) when children were in 5th grade. Average parent IQ was 113.5 (SD = 18.4, range = 80–149).

#### Child Peabody Picture Vocabulary Test

To examine the impact of early parent language input on later child brain imaging, above and beyond the child’s language skill at the time of imaging, we included a measure of children’s language skill (Peabody Picture Vocabulary Test, PPVT III; Dunn and Dunn, 1997), administered at 4th grade during the period when the imaging was done. The PPVT is a widely used measure of vocabulary comprehension with published norms. Average PPVT score was 113.61.

### Neuroimaging Procedure

Children were scanned in five waves from 9 to 16 years of age. As in other large-scale studies focusing on brain development during childhood, this age span was selected to capture a rapid period of brain development during late childhood and adolescence when high individual variability is observed (e.g., Sowell et al., 2001, 2002; Casey et al., 2018). For a detailed summary of the number of children that participated in each year and their age, see Table 1. Not all children participated in all scanning sessions and each child contributed 1–5 scans. Six children were scanned once, 3 children were scanned twice, 4 three times, 2 four times, and 8 five times. On average children were scanned 2.9 times, and we had a total of 83 scans. Although the sample size is modest (*n* = 23), it is important to highlight that, according to a recent review on neuroimaging studies on structural brain development, only 16 prior studies had on average more than 2 scans per participant, and only 3 prior studies had included three or more scans on average per participant (Vijayakumar et al., 2018).

**TABLE 1:** Descriptive statistics for child age at MRI scanning session.

	Age		
	*M* (SD)	Range	*n*
Year 1	9.31 (0.54)	8.66–10.29	16
Year 2	10.47 (0.60)	9.71–11.32	11
Year 3	11.44 (0.50)	10.79–12.19	13
Year 4	13.94 (0.59)	13.21–15.29	19
Year 5	15.97 (0.28)	15.48–16.40	14

*Descriptive statistics include mean age (M), standard deviation (SD), and sample size per year with valid measurements (n).*

### MRI Acquisition

The first to third waves of imaging data were acquired on a 3T Siemens Trio scanner with a 32-channel head-coil at Northwestern University’s Center for Translational Imaging in Chicago. A T1-weighted structural scan was acquired for each participant (1 mm × 1 mm × 1 mm resolution; sagittal acquisition). T1-weighted 3D spoiled gradient echo (MP-RAGE) sequences were obtained with TR = 2,300 ms, TE = 2.91 ms, flip angle = 9°, inversion time = 900 ms, and 256 contiguous slices (slice thickness = 1 mm, voxel size = 1 mm × 1 mm × 1 mm, matrix size = 256 × 256). The fourth to fifth waves of imaging data were acquired on a 3T Siemens Prisma Scanner with a 32-channel head-coil, also at the Northwestern University Center for Translational Imaging. A T1-weighted structural scan was acquired with a magnetization-prepared rapid gradient echo (MP-RAGE) sequence (TR = 2300 ms, TE = 1.86 ms, flip angle = 7°, Inversion Time = 1180 ms, 208 contiguous sagittal slices, slice thickness = 0.8 mm, voxel size = 0.8 mm × 0.8 mm × 0.8 mm, matrix size = 320 × 320). Head motion was minimized using foam padding around the head, and scanner noise was minimized with earplugs.

### Freesurfer Processing: Cortical Parcelation

Cortical reconstruction of white and pial surface models was performed using Freesurfer version 5.3.0^1^ (see Dale et al., 1999; Fischl et al., 1999). The cortical surface models were manually reviewed and edited for technical accuracy. We also performed quality assurance using the Freesurfer QA Toolbox v1.2. Sulcal and gyral structures were identified automatically (Fischl, 2004) and parcellated using the Destrieux cortical atlas for anatomical labeling (Destrieux et al., 2010). This parcelation scheme results in 148 cortical regions (74 per hemisphere). Cortical thickness was estimated as the average distance between the white and the pial surface reconstructions (Fischl and Dale, 2000).

Given our overall modest sample size, we focused on six ROIs that have been shown to play a particularly important role in language processing. Based on the previous literature on neurobiological basis of language development and our own work, which has found a relation between children’s own early language skills and their later cortical thickness in the children observed in this study (Asaridou et al., 2017), we examined cortical thickness in six ROIs in each hemisphere (12 regions in total): STG, STS, MTG, SMG, IFG (pars opercularis and pars triangularis) in each of the hemispheres (e.g., Price, 2010; Li et al., 2014).

### Statistical Analysis Plan

To address our research question, we built two sets of models. For the first set of models, we ran traditional, frequentist analysis of the data using linear mixed models. Given the sample size, we also performed the model fitting process under a Bayesian paradigm to complement the frequentist analyses (McNeish and Stapleton, 2016). The second set of analyses can be found in Supplementary Tables 1, 2 and Supplementary Figure 1. The direction of effects for parameters of interest was consistent across the frequentist and Bayesian models. For the frequentist approach, linear mixed models were built in R using the lmer package (R Core Team, 2017). The dependent variable was cortical thickness. We started with a parsimonious model of fixed effects including variables which, on theoretical grounds, we wanted to control for independent of effect size. These variables included a measure for parent SES composite, maternal IQ, sex, age, and an indicator for the fMRI scanner used (which changed after the 3rd scan). As measures for body size were unavailable, a mean thickness from 5 occipital regions of the brain (middle occipital gyrus, superior occipital, occipital pole, occipital sulcus, and parieto-occipital sulcus), typically not associated with auditory language processing, were used to control for brain size (Price, 2010). As we were interested in change in thickness over time, the minimum age value was subtracted from all ages, thus centering age at the beginning of the first scan. Similar to other longitudinal studies, this was done so that the zero-time point (the beginning of first scan) was included in the range of the model, and so that age-related coefficients could be interpreted as one-year increases in age from the onset of the scans. Further, by doing so, we can interpret the intercept term as cortical thickness at the beginning of the study. Additionally, exploratory analyses suggested that the largest difference in mean thickness between high and low language input groups (partitioned by the sign of the first principal component) occurred at younger ages. Setting the adjusted age to start at zero allowed this time to serve as a baseline for model covariates. Model covariates for IQ, language input composite, SES composite, mean occipital thickness were all centered and scaled.

Previous studies (e.g., Vijayakumar et al., 2016) indicate that change in brain thickness during the 9–16 year period, especially in the areas we focus on, could follow a quadratic pattern. Since our null hypothesis was that linguistic input does not have relate to brain thickness over time, we included a quadratic interaction between age and model covariates. Specifically, in addition to the hypothesized quadratic change in thickness over age, we attempted to account for the fact that linguistic input itself (as well as other covariates) may have a quadratic effect on cortical thickness. Inclusion of input for language, as well as quadratic interactions of all variables with age, were determined by Akaike information criterion (AIC). Random effects for the model were used to account for correlation between observations, and were selected based on the restricted maximum likelihood (REML) criterion. Random effects selected include random intercepts for subject, brain region, and laterality. A residuals analysis was performed on the final models to verify the assumption of normality for the model error. Reported *p*-values were computed with Satterthwaite approximation in the R package lmerTest (Kuznetsova et al., 2016).

## Results

### Descriptive Analyses

Parents showed variability in the quantity and complexity of language input at 18, 30, and 42 months (see Table 2). For example, some parents produced no decontextualized utterances at all; others produced over 600 during their 90-min visits. We included in this study only those children who took part in the MRI study. The numbers for the subsample who were included in the study were representative of the results based on the full sample discussed in other publications (e.g., Rowe, 2012). Parent input measures on our subsample at different time points were significantly correlated with each other, with correlations ranging from 0.19 to 0.74, and an average correlation of 0.48 – consistent with our work with the full sample showing that parents are relatively stable in their input over time (Silvey et al., 2021). See the Supplementary Materials for correlations between different time points separately (Supplementary Table 3).

**TABLE 2:** Descriptive statistics for parental input measures including mean (*M*), standard deviation (SD), and min–max range (*n* = 23).

	18 months	30 months	42 months
	
	*M* (SD) Range		
Word tokens	3.435 (2.176) 360–9.227	3.603 (1.825) 1.096–7.673	3.634 (1.923) 488–9.087
Decontextualized utterances	18.87 (22.23) 0–73	62.3 (67.01) 0–301	84.13 (133.09) 0–628
Rare words	25.57 (19.52) 1–83	30.09 (15.59) 10–76	37.35 (22.72) 6–100

Because the primary goal of the current study was to gather an overall view of children’s early language input, we used PCA to create a single composite measure of language input. The decision to focus on a composite input measure was further justified by the high degree of collinearity between the different measures. Measures of parental input were significantly correlated with each other (mean word tokens and decontextualized utterances, *r* = 0.81, *p* < 0.001; mean word tokens and rare words, *r* = 0.59, *p* < 0.001; mean decontextualized utterances and rare words, *r* = 0.45, *p* < 0.001). However, including rare words in the PCA decreased the variance explained by the first principal component to 32%, a net loss even after considering the addition of three measures (one for each time point), suggesting that including rare words increased noise that may, or may not, be related to our outcome of interest. We conducted analyses by combining mean word tokens and decontextualized utterances from the three time points into a single composite input score. The first principal component was highly correlated with each of these input measures and accounted for 62% of the total variance in linguistic input. Consequently, we focused on the principal component including word tokens and decontextualized utterances. We replicated the analysis with models using a principal component that also included rare word types. These models revealed similar results, though with a higher AIC, i.e., a worse fit. The results using the measure of parental input that also included rare words are included in the Supplementary Table 4.

### Linear Mixed Model Approach

**FIGURE 1:**
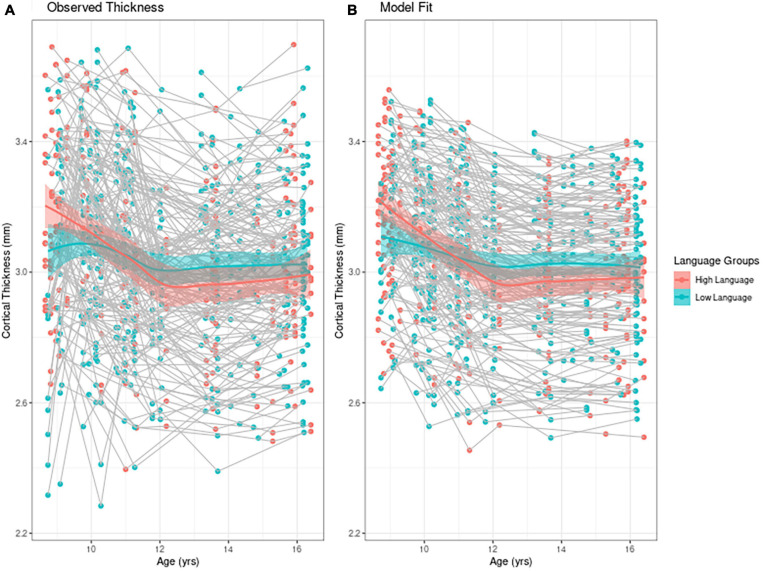
Individual trajectories of cortical thickness **(A)** mean observed cortical thickness by group and **(B)** model fit by group. For visualization purposes only, high and low language groups are separated by median language input PCA value. In both figures the solid line represents the Loess curve fit on the observation. The six children who participated in only one MRI session are represented with a single point in the figure.

To find the best fitting model for cortical thickness, we first present an empirical plot of children’s cortical thickness between 9 and 16 years. Figure 1A is a plot of the observed individual trajectories of cortical thickness for each participant. Please note that language input was measured continuously for the statistical analyses. For visualization purposes only, we divided the observations into high/low language input groups separated by the median language input PCA value. Superimposed on both of these plots is a solid line for each group representing the Loess curves fit to the values. Children who participated in only one MRI session (*n* = 6) are represented with a single point in the figure. We see that children with higher language input had higher values for cortical thickness than their lower language input peers, while also exhibiting steeper *change* within the time period observed. Figure 1B represents the model fit which we describe next.

To formally test the patterns observed, we ran linear mixed model analyses. Results are shown in Table 3. Considering our covariates first, we saw a non-linear effect with age on cortical thickness. Mean occipital thickness, as expected, was a significant predictor of thickness in our ROIs. Higher SES was associated with greater thickness overall, and SES moderated the relations of age to cortical thickness, where higher SES children had larger decreases in cortical thickness, compared to lower SES children. Female sex was both positively related to overall cortical thickness and negatively associated with change over time. Female sex was also positively related to the quadratic term. Mother IQ and scanner type did not predict cortical thickness when accounting for other factors in the model.

**TABLE 3:** Results of a linear mixed model analysis for the relationship between parental language input PCA (word tokens and decontextualized utterances) and cortical thickness.

	Estimate	Std. Error	95% L	95% U	*p*-value
Intercept	3.1692	0.1615	2.8527	3.4857	< 0.001*
Age	–0.0293	0.0188	–0.0662	0.0077	0.1208
Age^2^	0.0018	0.0024	–0.0029	0.0064	0.4593
Mean occipital thickness	0.0872	0.008	0.0716	0.1029	<0.001*
Scanner	0.0195	0.0283	–0.036	0.075	0.492
Mother IQ	–0.0009	0.0022	–0.0052	0.0033	0.6694
SES composite	0.0205	0.0271	–0.0327	0.0737	0.4572
SES composite × Age	–0.0061	0.0025	–0.011	–0.0012	0.0148*
Sex	0.1569	0.0667	0.0261	0.2877	0.0216*
Sex Age	–0.0762	0.0315	–0.1379	–0.0144	0.016*
Sex Age2	0.0072	0.0039	–0.0004	0.0148	0.0653
Language input PCA	0.068	0.0369	–0.0044	0.1404	0.069
Language input PCA × Age	–0.0378	0.0156	–0.0685	–0.0072	0.0158*
Language input PCA × Age^2^	0.0042	0.0019	0.0005	0.0079	0.0274*

We controlled for the following covariates: child age, mean occipital thickness, sex, scanner type, mother IQ, and family SES. Inferential statistics include estimate, standard error, 95% CI upper and lower limit, and p-value.

*p < 0.05.

Particularly relevant to our main question, we saw that language input interacted quadratically with age. Specifically, the covariates indicate a concave upward parabola for children with greater language input during the early years than for children with less language input. This trend suggests that children with high language input had overall higher cortical thickness at the beginning of the observed time period, i.e., around 9 years of age. The signs and effect sizes of the linear and quadratic terms suggest that children with higher language input experienced larger decreases in cortical thickness in the 9–16 age range, compared to children with lower language input, who exhibited a more attenuated change. Although our sample size is modest, the direction of effects for parameters of interest is consistent across the frequentist and Bayesian models (see Supplementary Table 5). Further, the directions of effects for parameters for age are also consistent with prior studies (Vijayakumar et al., 2016, 2018). Importantly, the results remained unchanged when analyses were repeated on the subsample of 17 children who had at least two or more scans and we could directly assess change over time (see Supplementary Materials 5). Since SES and language input composite are both centered and scaled, it is possible to compare the effect of the two factors in the model. The estimates suggest that the effect size of language input and age on cortical thickness is comparable to (slightly larger) than the effect size of SES on cortical thickness. Finally, to examine the specificity of the relations of later input to later child cortical thickness, we included children’s PPVT scores as a covariate in our model; the results were unchanged (see Supplementary Table 6).

### Region-Specific Relations

Given our modest sample size, we refrain from making strong conclusions about region-specific effects. For completeness, we report exploratory analyses including intercept, linear, and quadratic terms for each ROI as fixed factors. The values for non-region relevant covariates, such as age, SES, and sex matched the previous main models. Previous analyses did not reveal strong laterality differences and thus cortical thickness for the left and right were averaged per region. IFG pars opercularis showed the strongest relation to language input and STG and STS revealed non-significant trending associations. No significant relations were observed in other regions such as IFG pars triangularis or SMG. For these former three regions, as in the main model, language input was positively related to thickness at baseline, negative to slope and positive to quadratic term. In other words, children with greater early input had a higher intercept (indicating higher cortical thickness at the beginning of the study), and a steeper change over the observed time period than children with less early input. The tentative conclusion that language input might have a particular impact on IFG, STG, and STS is supported by previous evidence (e.g., Romeo et al., 2018a). See Supplementary Materials for region-specific linear mixed-model analysis (Supplementary Table 7), region-specific cortical thickness change trajectories (Supplementary Figure 2), and region-specific Bayesian analysis (Supplementary Table 2).

## Discussion

Our results reveal, for the first time, that early parental language input prior to school predicts *changes* in children’s language-related cortical structures during the school years in mid-adolescence. Cortical thickness decreases during childhood, particularly from mid-childhood to adolescence (Mills et al., 2014; Wierenga et al., 2014; Vijayakumar et al., 2016; Tamnes et al., 2017). Even though there is a general decline in thickness, the trajectory of change displays substantial individual variability, and the trajectories vary by region. Vijayakumar et al. (2016) reported a negative quadratic pattern of cortical thickness change in temporal areas, which is similar to the pattern observed here. Individual variability is largest in temporal and frontal regions across the lifespan (Frangou et al., 2020). These structural variations can be linked to a wide range of child internal factors. For example, Asaridou et al. (2017) showed, in the sample studied here, that differences in children’s early language development predict differences in later brain structure. The structural variations can also be linked to a wide range of experiential factors. For example, SES and parental cognitive stimulation predict variability in child brain regions supporting language processing (e.g., Luby et al., 2013; Merz et al., 2020). To the extent that previous studies explore the relations between parental language input and child brain structure, they focused on *concurrent* relations between input and brain structure. In contrast, we examined *predictive* relations between early parental language input and later child brain structure. The strength of our approach is that we modeled *change* in later brain structure using longitudinal data with multiple observations.

We found unique relations between early parental input and *change* in later child cortical thickness, which were stronger than relations between early parental input and the average level of later child cortical thickness. Finding stronger relations between parent input and change over time in child brain structure than to values at a single time-point dovetails with previous work showing that the *trajectory* of cortical thickness, rather than its value at a given time point, is a good index of individual variability in performance (Sowell et al., 2001). We found that the greater the early parental language input, the steeper the change in child cortical thickness years later. In other words, change was slower for children at the lower end of the parent input continuum. Our findings are also consistent with studies showing extended growth trajectories in children from higher SES families (Hanson et al., 2013), continued cortical thinning in children from higher SES families throughout late adolescence, and early plateauing in children from lower SES families (e.g., Piccolo et al., 2016), which is considered a sign of accelerated development in children from lower SES families (LeWinn et al., 2017). With respect to cortical thickness, children from higher SES backgrounds show steady age-related decreases, particularly in regions related to language processing (e.g., left STG); in contrast, children from lower SES backgrounds begin to plateau during late adolescence (Piccolo et al., 2016; McDermott et al., 2019). We extend previous work by identifying, for the first time, a direct measure – early parental language input – that predicts later change in child cortical thickness, over and above SES.

Why might children who have been exposed to a higher quantity and complexity of language input early in development exhibit continued change in cortical thickness, whereas low input children plateau? Certain enriching experiences might keep the window for structural brain development open, allowing for additional cortical thinning. In contrast, developmental thinning might be sped up for individuals who are not as frequently exposed to enriching experiences, resulting in an earlier-closing window and less thinning overall. Literature on severe environmental adversity, such as traumatic childhood experiences, supports the notion that damaging early life experiences can derail brain development, specifically leading to accelerated maturation and narrower windows of plasticity (Gur et al., 2019; Miskolczki et al., 2019). Here, we focused on only the role of enriching experiences and variability within the normative range. However, within the normative range, our results suggest a comparable profile of extended change for children exposed to richer experiences, and of restricted change for children with more impoverished input. Future work is needed to explore whether negative and positive experiences are part of the same continuum with respect to brain development. Another possible explanation for the differences we find between children coming from homes that provide high versus low levels of cognitive enrichment is that children exposed to more language input might have resources to spare, as evidenced by their overall greater cortical thickness early in development, which might lead to continued thinning. In other words, more parental input might lead to thicker cortex to begin with, which then supports more protracted thinning. Overall, the computational properties of a network might be better revealed by considering its developmental origins *and* change over time together (Dündar-Coecke and Thomas, 2019).

Three developmental theories have been proposed to explain apparent cortical thinning in the age range examined: pruning, myelination and cortical morphology, but not to neuron generation or loss (Vandekar et al., 2015; Vijayakumar et al., 2016). Synapse elimination, pruning and myelination continues well into adulthood (Huttenlocher, 2009). Important underlying cellular changes include changes in the number of neurons in a cortical column, the amount of glial and capillary support, and dendritic branching (Rakic, 1988, 2009). More recent evidence supports the hypothesis that the cortical thinning during childhood is primarily due to increased myelination. The observed thinning is considered to be due to increased myelination altering the contrast between gray and white matter in MRI images, which in turn affects the apparent cortical boundary (Natu et al., 2019). Experiential factors have been shown to predict myelination (e.g., Hensch, 2004; Knudsen, 2004). Myelination is also important for concluding of periods of plasticity (Hensch, 2004; Hensch and Bilimoria, 2012). Taken together, reduced environmental stimulation might be associated with early narrowing of plasticity associated with overall lower myelination which might then result in smaller subsequent changes in cortical thickness, whereas higher input might keep windows of plasticity longer and thus might be associated with larger subsequent changes (Tooley et al., 2021).

The behavioral mechanisms by which early parental input relates to later child brain development remains an open question. One possibility is that simply being exposed to rich language input influences efficiency of language processing (e.g., Fernald et al., 2013), which, in turn, is associated with changes at the neural level. Another possibility is that, when parents produce rich language, children engage in rich conversations and it is *children* producing language that is associated with differences in brain structure (Romeo et al., 2018a). The two possibilities are not mutually exclusive, and it is also possible that the relation between early parental input and later child behavioral and neurological development varies depending on the specific brain region considered. We did not have early measures of children’s general cognitive development, such as their memory or attention. Measures of general cognitive development would be needed to establish the specificity of the relation between early parental language input and later child neurological development, and to explore whether parental input might relate to later child outcomes via broader aspects of cognitive development. Although we emphasize the role of parental input, children are active participants in this interaction and might drive the input in different ways. Our recent work presents novel statistical models that account for the contribution of the child in eliciting parental input (Silvey et al., 2021), which we are currently applying to neuroimaging data. Finally, whether it is early parental input that predicts later change in child brain structures (which would be consistent with sensitive period hypotheses, Newport et al., 2001), or whether parents must continue to provide rich input to their children to trigger later change needs to be examined in future studies.

A number of limitations should be considered when interpreting our results. First, our findings are correlational. Although we accounted for important covariates, such as parental education and IQ, we cannot make claims about causation. Second, we have a modest sample size, which limits our ability to derive strong conclusions. We also observed multicollinearity between some covariates. For example, children with greater language input were more likely to be male and from higher SES homes, giving us few observations with which to isolate either of these covariates. Despite the uncertainty in our models (frequentist and Bayesian), the parameter fits from the observed data largely match the background literature and support our hypotheses. Further, we focused on a single factor, parental language input, following previous work emphasizing the role of early parental input in predicting later child behavioral outcomes, and also its role in predicting structural differences in brain areas subserving language processing at the same age. One could argue that language input might be correlated with other factors that predict brain structure – some of these include general cognitive stimulation, toxins, sleep differences, and even stress and glucocorticoids during pregnancy (Kaufman and Charney, 2001; Davis et al., 2013). We attempted to account for other general differences in children’s environments by controlling for overall parental SES, as well as parental IQ. However, we cannot rule out the possibility that there were unique but related factors that contributed to differences in later child brain structure. Future work is needed to compare and contrast the role of other experiential factors on later changes in child cortical structure. Finally, although our study might be underpowered and recent reports suggest that replicable brain-behavior correlations with fMRI may require larger sample sizes (Yarkoni and Braver, 2010; Marek et al., 2020) than the one used here, our results are valuable in that they generate hypotheses to be tested with larger samples.

In sum, our study leverages a unique longitudinal dataset combining naturalistic observations of parent–child interactions and structural neuroimaging measures during a period spanning 15 years. For the first time, we show that early parental language input prior to school predicts changes in child cortical structure in mid-adolescence, over and above the contributions of SES or parental IQ. Our results are consistent with previous work examining SES-related differences in brain structure, and move the literature forward by contributing to our understanding of the mechanisms underlying individual differences in brain development. Pinpointing specific experiential factors that predict brain structure has the potential to inform prevention and intervention strategies designed to draw upon and integrate early home support.

## Data Availability Statement

The raw data supporting the conclusions of this article will be made available by the authors, without undue reservation.

## Ethics Statement

The studies involving human participants were reviewed and approved by the University of Chicago BSD/UCMC Institutional Review Boards and the Office of Research at the University of California, Irvine. Written informed consent to participate in this study was provided by the participants’ legal guardian/next of kin.

## Author Contributions

SS and SG-M conceived the longitudinal project. ÖD-L and SA were involved in the neuroimaging data collection and analysis. ÖD-L was involved in parental language input coding and drafted the manuscript. ÖD-L and CN analyzed the data for the current manuscript in consultation with SA, SS, and SG-M. All authors critically edited, extensively contributed to the project, and approved the final submitted version of the manuscript.

## Conflict of Interest

The authors declare that the research was conducted in the absence of any commercial or financial relationships that could be construed as a potential conflict of interest.

## Publisher’s Note

All claims expressed in this article are solely those of the authors and do not necessarily represent those of their affiliated organizations, or those of the publisher, the editors and the reviewers. Any product that may be evaluated in this article, or claim that may be made by its manufacturer, is not guaranteed or endorsed by the publisher.

## Footnotes

1 See http://surfer.nmr.mgh.harvard.edu/ for details on the Freesurfer surface-based pipeline.

## References

[B1] Anderson B. J. Li X. Alcantara A. A. Isaacs K. R. Black J. E. Greenough W. T. (1994). Glial hypertrophy is associated with synaptogenesis following motor-skill learning, but not with angiogenesis following exercise. *Glia* 11 73–80. 10.1002/glia.440110110 7520887

[B2] Asaridou S. S. Demir-Lira Ö. E. Goldin-Meadow S. Small S. L. (2017). The pace of vocabulary growth during preschool predicts cortical structure at school age. *Neuropsychologia* 98 13–23. 10.1016/j.neuropsychologia.2016.05.018 27212056 PMC5116280

[B3] Avants B. B. Hackman D. A. Betancourt L. M. Lawson G. M. Hurt H. Farah M. J. (2015). Relation of childhood home environment to cortical thickness in late adolescence: specificity of experience and timing. *PLoS One* 10:e0138217. 10.1371/journal.pone.0138217 26509809 PMC4624931

[B4] Badcock N. A. Bishop D. V. Hardiman M. J. Barry J. G. Watkins K. E. (2012). Co-localisation of abnormal brain structure and function in specific language impairment. *Brain Lang.* 120 310–320. 10.1016/j.bandl.2011.10.006 22137677 PMC3315677

[B5] Barnes J. (2011). The influence of child-directed speech in early trilingualism. *Int. J. Multiling.* 8 42–62. 10.1080/14790711003671861

[B6] Beals D. Tabors P. (1995). Arboretum, bureaucratic and carbohydrates: preschoolers’ exposure to rare vocabulary at home. *First Lang.* 15 57–76.

[B7] Black J. E. Isaacs K. R. Anderson B. J. Alcantara A. A. Greenough W. T. (1990). Learning causes synaptogenesis, whereas motor activity causes angiogenesis, in cerebellar cortex of adult rats. *Proc. Natl. Acad. Sci. U.S.A.* 87 5568–5572. 10.1073/pnas.87.14.5568 1695380 PMC54366

[B8] Bleses D. Makransky G. Dale P. S. Højen A. Ari B. A. (2016). Early productive vocabulary predicts academic achievement 10 years later. *Appl. Psycholinguist.* 37 1461–1476. 10.1017/s0142716416000060

[B9] Cartmill E. A. Armstrong B. F. Gleitman L. R. Goldin-Meadow S. Medina T. N. Trueswell J. C. (2013). Quality of early parent input predicts child vocabulary 3 years later. *Proc. Natl. Acad. Sci. U.S.A.* 110 11278–11283. 10.1073/pnas.1309518110 23798423 PMC3710871

[B10] Casey B. J. Cannonier T. Conley M. I. Cohen A. O. Barch D. M. Heitzeg M. M. (2018). The adolescent brain cognitive development (ABCD) study: imaging acquisition across 21 sites. *Dev. Cogn. Neurosci.* 32 43–54. 29567376 10.1016/j.dcn.2018.03.001 PMC5999559

[B11] Chall J. S. Dale E. (1995). *Readability Revisited: The New Dale-Chall Readability Formula.* Cambridge: Brookline Books.

[B12] Dale A. M. Fischl B. Sereno M. I. (1999). Cortical surface-based analysis I. Segmentation and surface reconstruction. *Neuroimage* 9 179–194. 9931268 10.1006/nimg.1998.0395

[B13] Dale E. Chall J. S. (1948). A formula for predicting readability: instructions. *Educ. Res. Bull.* 27 37–54.

[B14] Davis E. P. Sandman C. A. Buss C. Wing D. A. Head K. (2013). Fetal glucocorticoid exposure is associated with preadolescent brain development. *Biol. Psychiatry* 74 647–655. 10.1016/j.biopsych.2013.03.009 23611262 PMC3985475

[B15] Davis M. H. Gaskell M. G. (2009). A complementary systems account of word learning: neural and behavioural evidence. *Philos. Trans. R. Soc. B Biol. Sci.* 364 3773–3800. 10.1098/rstb.2009.0111 19933145 PMC2846311

[B16] Demir Ö. E. Rowe M. L. Heller G. Goldin-Meadow S. Levine S. C. (2015). Vocabulary, syntax, and narrative development in typically developing children and children with early unilateral brain injury: early parental talk about the “there-and-then” matters. *Dev. Psychol.* 51 161–175. 10.1037/a0038476 25621756 PMC4307606

[B17] Demir-Lira Ö. E. Applebaum L. R. Goldin-Meadow S. Levine S. C. (2019). Parents’ early book reading to children: relation to children’s later language and literacy outcomes controlling for other parent language input. *Dev. Sci.* 22:e12764. 10.1111/desc.12764 PMC6927670 30325107

[B18] Destrieux C. Fischl B. Dale A. Halgren E. (2010). Automatic parcellation of human cortical gyri and sulci using standard anatomical nomenclature. *Neuroimage* 53 1–15. 10.1016/j.neuroimage.2010.06.010 20547229 PMC2937159

[B19] Diamond M. C. Krech D. Rosenzweig M. R. (1964). The effects of an enriched environment on the histology of the rat cerebral cortex. *J. Comp. Neurol.* 123 111–119. 10.1002/cne.901230110 14199261

[B20] Duncan G. J. Dowsett C. J. Claessens A. Magnuson K. Huston A. C. Klebanov P. (2007). School readiness and later achievement. *Dev. Psychol.* 43 1428–1446. 18020822 10.1037/0012-1649.43.6.1428

[B21] Duncan G. J. Magnuson K. (2012). Socioeconomic status and cognitive functioning: moving from correlation to causation. *Wiley Interdiscip. Rev. Cogn. Sci.* 3 377–386. 10.1002/wcs.1176 26301469

[B22] Dündar-Coecke S. Thomas M. S. C. (2019). “Modelling socioeconomic effects on the development of brain and behaviour,” in *Cognitive Science*, eds Goel A. Seifert C. Freksa C. (Montreal: The Cognitive Science Society), 1676–1682.

[B23] Dunn L. M. Dunn L. M. (1997). *Peabody Picture Vocabulary Test–III.* Circle Pines, MN: American Guidance Service.

[B24] Fedorenko E. Thompson-Schill S. L. (2014). Reworking the language network. *Trends Cogn. Sci.* 18 120–126. 10.1016/j.tics.2013.12.006 24440115 PMC4091770

[B25] Fenson L. Dale P. S. Reznick J. S. Bates E. Thal D. J. Pethick S. J. (1994). Variability in early communicative development. *Monogr. Soc. Res. Child Dev.* 59 1–173. 10.4324/9781315111322-1 7845413

[B26] Fernald A. Marchman V. A. Weisleder A. (2013). SES differences in language processing skill and vocabulary are evident at 18 months. *Dev. Sci.* 16 234–248. 10.1111/desc.12019 23432833 PMC3582035

[B27] Fischl B. (2004). Automatically parcellating the human cerebral cortex. *Cereb. Cortex* 14 11–22. 10.1093/cercor/bhg087 14654453

[B28] Fischl B. Dale A. M. (2000). Measuring the thickness of the human cerebral cortex from magnetic resonance images. *Proc. Natl. Acad. Sci. U.S.A.* 97 11050–11055. 10.1073/pnas.200033797 10984517 PMC27146

[B29] Fischl B. Sereno M. I. Dale A. M. (1999). Cortical surface-based analysis: II: inflation, flattening, and a surface-based coordinate system. *Neuroimage* 9 195–207. 10.1006/nimg.1998.0396 9931269

[B30] Frangou S. Modabbernia A. Doucet G. E. Papachristou E. Williams S. C. Agartz I. (2020). Cortical Thickness Trajectories across the Lifespan: Data from 17,075 healthy individuals aged 3–90 years. *BioRxiv.* 10.1002/hbm.25364 PMC8675431 33595143

[B31] Friederici A. D. Gierhan S. M. (2013). The language network. *Curr. Opin. Neurobiol.* 23 250–254. 23146876 10.1016/j.conb.2012.10.002

[B32] Goldin-Meadow S. Levine S. C. Hedges L. V. Huttenlocher J. Raudenbush S. W. Small S. L. (2014). New evidence about language and cognitive development based on a longitudinal study: hypotheses for intervention. *Am. Psychol.* 69 588–599. 10.1037/a0036886 24911049 PMC4159405

[B33] Gullick M. M. Demir-Lira Ö. E. Booth J. R. (2016). Reading skill–fractional anisotropy relationships in visuospatial tracts diverge depending on socioeconomic status. *Dev. Sci.* 19 673–685. 10.1111/desc.12428 27412229 PMC5995108

[B34] Gur R. E. Moore T. M. Rosen A. F. Barzilay R. Roalf D. R. Calkins M. E. (2019). Burden of environmental adversity associated with psychopathology, maturation, and brain behavior parameters in youths. *JAMA Psychiatry* 76 966–975. 10.1001/jamapsychiatry.2019.0943 31141099 PMC6547104

[B35] Hair N. L. Hanson J. L. Wolfe B. L. Pollak S. D. (2015). Association of child poverty, brain development, and academic achievement. *JAMA Pediatr.* 169 822–829. 10.1001/jamapediatrics.2015.1475 26192216 PMC4687959

[B36] Hanson J. L. Hair N. Shen D. G. Shi F. Gilmore J. H. Wolfe B. L. (2013). Family poverty affects the rate of human infant brain growth. *PLoS One* 8:e80954. 10.1371/journal.pone.0080954 24349025 PMC3859472

[B37] Hart B. Risley T. R. (1995). *Meaningful Differences in the Everyday Experience of Young American Children.* Baltimore, MD: Paul H Brookes Publishing.

[B38] Hensch T. K. (2004). Critical period regulation. *Annu. Rev. Neurosci.* 27 549–579. 10.1146/annurev.neuro.27.070203.144327 15217343

[B39] Hensch T. K. Bilimoria P. M. (2012). Re-opening windows: manipulating critical periods for brain development. *Cerebrum* 2012:11. PMC3574806 23447797

[B40] Hickok G. Poeppel D. (2007). The cortical organization of speech processing. *Nat. Rev. Neurosci.* 8 393–402. 10.1038/nrn2113 17431404

[B41] Hoff E. (2003). The specificity of environmental influence: socioeconomic status affects early vocabulary development via maternal speech. *Child Dev.* 74 1368–1378. 10.1111/1467-8624.00612 14552403

[B42] Huttenlocher J. Haight W. Bryk A. Seltzer M. Lyons T. (1991). Early vocabulary growth: relation to language input and gender. *Dev. Psychol.* 27 236–248. 10.1037/0012-1649.27.2.236

[B43] Huttenlocher J. Vasilyeva M. Cymerman E. Levine S. (2002). Language input and child syntax. *Cognit. Psychol.* 45 337–374. 10.1016/s0010-0285(02)00500-5 12480478

[B44] Huttenlocher P. R. (2009). *Neural Plasticity.* Cambridge, MA: Harvard University Press.

[B45] Jednoróg K. Altarelli I. Monzalvo K. Fluss J. Dubois J. Billard C. (2012). The influence of socioeconomic status on children’s brain structure. *PLoS One* 7:e42486. 10.1371/journal.pone.0042486 22880000 PMC3411785

[B46] Kaufman J. Charney D. (2001). Effects of early stress on brain structure and function: implications for understanding the relationship between child maltreatment and depression. *Dev. Psychopathol.* 13 451–471. 10.1017/s0954579401003030 11523843

[B47] Kleim J. A. Lussnig E. Schwarz E. R. Comery T. A. Greenough W. T. (1996). Synaptogenesis and Fos expression in the motor cortex of the adult rat after motor skill learning. *J. Neurosci.* 16 4529–4535. 10.1523/jneurosci.16-14-04529.1996 8699262 PMC6578852

[B48] Knudsen E. I. (2004). Sensitive periods in the development of the brain and behavior. *J. Cogn. Neurosci.* 16 1412–1425. 10.1162/0898929042304796 15509387

[B49] Kuznetsova A. Brockhoff P. B. Christensen R. H. B. (2016). *Tests in Linear Mixed Effects Models Version. Cran.*

[B50] Lee J. C. Dick A. S. Tomblin J. B. (2020). Altered brain structures in the dorsal and ventral language pathways in individuals with and without developmental language disorder (DLD). *Brain Imaging Behav.* 14 2569–2586. 10.1007/s11682-019-00209-1 31933046 PMC7354888

[B51] LeWinn K. Z. Sheridan M. A. Keyes K. M. Hamilton A. McLaughlin K. A. (2017). Sample composition alters associations between age and brain structure. *Nat. Commun.* 8:874. 10.1038/s41467-017-00908-7 PMC5638928 29026076

[B52] Li P. Legault J. Litcofsky K. A. (2014). Neuroplasticity as a function of second language learning: anatomical changes in the human brain. *Cortex* 58 301–324. 10.1016/j.cortex.2014.05.001 24996640

[B53] Luby J. Belden A. Botteron K. Marrus N. Harms M. P. Babb C. (2013). The effects of poverty on childhood brain development: the mediating effect of caregiving and stressful life events. *JAMA Pediatr.* 167 1135–1142. 10.1001/jamapediatrics.2013.3139 24165922 PMC4001721

[B54] Mackey A. P. Finn A. S. Leonard J. A. Jacoby-Senghor D. S. West M. R. Gabrieli C. F. (2015). Neuroanatomical correlates of the income-achievement gap. *Psychol. Sci.* 26 925–933. 10.1177/0956797615572233 25896418 PMC4458190

[B55] Marchman V. A. Fernald A. (2008). Speed of word recognition and vocabulary knowledge in infancy predict cognitive and language outcomes in later childhood. *Dev. Sci.* 11 F9–F16. 18466367 10.1111/j.1467-7687.2008.00671.x PMC2905590

[B56] Marek S. Tervo-Clemmens B. Calabro F. J. Montez D. F. Kay B. P. Hatoum A. S. (2020). Towards reproducible brain-wide association studies. *BioRxiv.*

[B57] McDermott C. L. Seidlitz J. Nadig A. Liu S. Clasen L. S. Blumenthal J. D. (2019). Longitudinally mapping childhood socioeconomic status associations with cortical and subcortical morphology. *J. Neurosci.* 39 1365–1373. 10.1523/jneurosci.1808-18.2018 30587541 PMC6381251

[B58] McNeish D. M. Stapleton L. M. (2016). The effect of small sample size on two-level model estimates: a review and illustration. *Educ. Psychol. Rev.* 28 295–314. 10.1007/s10648-014-9287-x

[B59] Merz E. C. Maskus E. A. Melvin S. A. He X. Noble K. G. (2020). Socioeconomic disparities in language input are associated with children’s language-related brain structure and reading skills. *Child Dev.* 91 846–860. 10.1111/cdev.13239 30919945 PMC6765463

[B60] Mills K. L. Goddings A. L. Herting M. M. Meuwese R. Blakemore S. J. Crone E. A. (2016). Structural brain development between childhood and adulthood: convergence across four longitudinal samples. *Neuroimage* 141 273–281. 10.1016/j.neuroimage.2016.07.044 27453157 PMC5035135

[B61] Mills K. L. Lalonde F. Clasen L. S. Giedd J. N. Blakemore S. J. (2014). Developmental changes in the structure of the social brain in late childhood and adolescence. *Soc. Cogn. Affect. Neurosci.* 9 123–131. 10.1093/scan/nss113 23051898 PMC3871734

[B62] Miskolczki C. Halász J. Mikics É (2019). Changes in neuroplasticity following early-life social adversities: the possible role of brain-derived neurotrophic factor. *Pediatr. Res.* 85 225–233. 10.1038/s41390-018-0205-7 30341412

[B63] Mutlu A. K. Schneider M. Debbané M. Badoud D. Eliez S. Schaer M. (2013). Sex differences in thickness, and folding developments throughout the cortex. *Neuroimage* 82 200–207. 10.1016/j.neuroimage.2013.05.076 23721724

[B64] Natu V. S. Gomez J. Barnett M. Jeska B. Kirilina E. Jaeger C. (2019). Apparent thinning of human visual cortex during childhood is associated with myelination. *Proc. Nat. Acad. Sci.* 116 20750–20759. 10.1073/pnas.1904931116 31548375 PMC6789966

[B65] Newport E. L. Bavelier D. Neville H. J. (2001). “Critical thinking about critical periods: perspectives on a critical period for language acquisition,” in *Language, Brain, and Cognitive Development: Essays in Honor of Jacques Mehler*, ed. E. Dupoux (MIT Press), 481–502.

[B66] Noble K. G. Houston S. M. Brito N. H. Bartsch H. Kan E. Kuperman J. M. (2015). Family income, parental education and brain structure in children and adolescents. *Nat. Neurosci.* 18 773–778. 10.1038/nn.3983 25821911 PMC4414816

[B67] Noble K. G. Houston S. M. Kan E. Sowell E. R. (2012). Neural correlates of socioeconomic status in the developing human brain. *Dev. Sci.* 15 516–527. 10.1111/j.1467-7687.2012.01147.x 22709401 PMC6554027

[B68] Oldfield R. C. (1971). The assessment and analysis of handedness: the Edinburgh inventory. *Neuropsychologia* 9 97–113. 10.1016/0028-3932(71)90067-4 5146491

[B69] Piccolo L. R. Merz E. C. He X. Sowell E. R. Noble K. G. Pediatric Imaging (2016). Age-related differences in cortical thickness vary by socioeconomic status. *PLoS One* 11:e0162511. 10.1371/journal.pone.0162511 27644039 PMC5028041

[B70] Price C. J. (2010). The anatomy of language: a review of 100 fMRI studies published in 2009. *Ann. N. Y. Acad. Sci.* 1191 62–88. 10.1111/j.1749-6632.2010.05444.x 20392276

[B71] Price C. J. (2012). A review and synthesis of the first 20 years of PET and fMRI studies of heard speech, spoken language and reading. *Neuroimage* 62 816–847. 10.1016/j.neuroimage.2012.04.062 22584224 PMC3398395

[B72] R Core Team (2017). *R: A Language and Environment for Statistical Computing. R Foundation for Statistical Computing.* Vienna: R Core Team.

[B73] Raizada R. D. Richards T. L. Meltzoff A. Kuhl P. K. (2008). Socioeconomic status predicts hemispheric specialisation of the left inferior frontal gyrus in young children. *Neuroimage* 40 1392–1401. 10.1016/j.neuroimage.2008.01.021 18308588 PMC2679945

[B74] Rakic P. (1988). Specification of cerebral cortical areas. *Science* 241 170–176. 10.1126/science.3291116 3291116

[B75] Rakic P. (2009). Evolution of the neocortex: a perspective from developmental biology. *Nat. Rev. Neurosci.* 10 724–735. 10.1038/nrn2719 19763105 PMC2913577

[B76] Raznahan A. Shaw P. Lalonde F. Stockman M. Wallace G. L. Greenstein D. (2011). How does your cortex grow? *J. Neurosci.* 31 7174–7177. 21562281 10.1523/JNEUROSCI.0054-11.2011 PMC3157294

[B77] Ribeiro P. F. Ventura-Antunes L. Gabi M. Mota B. Grinberg L. T. Farfel J. M. (2013). The human cerebral cortex is neither one nor many: neuronal distribution reveals two quantitatively different zones in the gray matter, three in the white matter, and explains local variations in cortical folding. *Front. Neuroanat.* 7:28. 10.3389/fnana.2013.00028 24032005 PMC3759024

[B78] Rodríguez-Fornells A. Cunillera T. Mestres-Missé A. de Diego-Balaguer R. (2009). Neurophysiological mechanisms involved in language learning in adults. *Philos. Trans. R. Soc. B Biol. Sci.* 364 3711–3735. 10.1098/rstb.2009.0130 19933142 PMC2846313

[B79] Romeo R. R. Leonard J. A. Robinson S. T. West M. R. Mackey A. P. Rowe M. L. (2018a). Beyond the 30-million-word gap: children’s conversational exposure is associated with language-related brain function. *Psychol. Sci.* 29 700–710. 10.1177/0956797617742725 29442613 PMC5945324

[B80] Romeo R. R. Segaran J. Leonard J. A. Robinson S. T. West M. R. Mackey A. P. (2018b). Language exposure relates to structural neural connectivity in childhood. *J. Neurosci.* 38 7870–7877. 10.1523/jneurosci.0484-18.2018 30104336 PMC6125810

[B81] Rowe M. (2008). Child-directed speech: relation to socioeconomic status, knowledge of child development and child vocabulary skill. *J. Child Lang.* 35 185–205. 10.1017/s0305000907008343 18300434

[B82] Rowe M. (2012). A longitudinal investigation of the role of quantity and quality of child-directed speech in vocabulary development. *Child Dev.* 83 1762–1774. 10.1111/j.1467-8624.2012.01805.x 22716950 PMC3440540

[B83] Rowe M. L. Goldin-Meadow S. (2009). Differences in early gesture explain SES disparities in child vocabulary size at school entry. *Science* 323 951–953. 10.1126/science.1167025 19213922 PMC2692106

[B84] Rowe M. L. Levine S. C. Fisher J. A. Goldin-Meadow S. (2009). Does linguistic input play the same role in language learning for children with and without early brain injury? *Dev. Psychol.* 45:90. 10.1037/a0012848 19209993 PMC2643358

[B85] Rowe M. L. Raudenbush S. W. Goldin-Meadow S. (2012). The pace of vocabulary growth helps predict later vocabulary skill. *Child Dev.* 83 508–525. 10.1111/j.1467-8624.2011.01710.x 22235920 PMC3262592

[B86] Rowe M. L. Snow C. E. (2020). Analyzing input quality along three dimensions: interactive, linguistic, and conceptual. *J. Child Lang.* 47 5–21. 10.1017/s0305000919000655 31668157

[B87] Shneidman L. A. Arroyo M. E. Levine S. C. Goldin-Meadow S. (2013). What counts as effective input for word learning? *J. Child Lang.* 40:672. 10.1017/s0305000912000141 22575125 PMC3445663

[B88] Silvey C. Demir-Lira Ö. E. Goldin-Meadow S. Raudenbush S. W. (2021). Effects of time-varying parent input on children’s language outcomes differ for vocabulary and syntax. *Psychol. Sci.* 32 536–548. 10.1177/0956797620970559 33720801 PMC8726591

[B89] Sowell E. R. Delis D. Stiles J. Jernigan T. L. (2001). Improved memory functioning and frontal lobe maturation between childhood and adolescence: a structural MRI study. *J. Int. Neuropsychol. Soc.* 7 312–322. 10.1017/s135561770173305x 11311032

[B90] Sowell E. R. Peterson B. S. Thompson P. M. Welcome S. E. Henkenius A. L. Toga A. W. (2003). Mapping cortical change across the human life span. *Nat. Neurosci.* 6 309–315. 10.1038/nn1008 12548289

[B91] Sowell E. R. Trauner D. A. Gamst A. Jernigan T. L. (2002). Development of cortical and subcortical brain structures in childhood and adolescence: a structural MRI study. *Dev. Med. Child Neurol.* 44 4–16. 10.1111/j.1469-8749.2002.tb00253.x 11811649

[B92] Tamnes C. K. Herting M. M. Goddings A. L. Meuwese R. Blakemore S. J. Dahl R. E. (2017). Development of the cerebral cortex across adolescence: a multisample study of inter-related longitudinal changes in cortical volume, surface area, and thickness. *J. Neurosci.* 37 3402–3412. 10.1523/jneurosci.3302-16.2017 28242797 PMC5373125

[B93] Tooley U. A. Bassett D. S. Mackey A. P. (2021). Environmental influences on the pace of brain development. *Nat. Rev. Neurosci.* 22 372–384. 10.1038/s41583-021-00457-5 33911229 PMC8081006

[B94] Vandekar S. N. Shinohara R. T. Raznahan A. Roalf D. R. Ross M. DeLeo N. (2015). Topologically dissociable patterns of development of the human cerebral cortex. *J. Neurosci.* 35 599–609. 10.1523/jneurosci.3628-14.2015 25589754 PMC4293413

[B95] Vijayakumar N. Allen N. B. Youssef G. Dennison M. Yücel M. Simmons J. G. (2016). Brain development during adolescence: a mixed-longitudinal investigation of cortical thickness, surface area, and volume. *Hum. Brain Mapp.* 37 2027–2038. 10.1002/hbm.23154 26946457 PMC6867680

[B96] Vijayakumar N. Mills K. L. Alexander-Bloch A. Tamnes C. K. Whittle S. (2018). Structural brain development: a review of methodological approaches and best practices. *Dev. Cogn. Neurosci.* 33 129–148. 10.1016/j.dcn.2017.11.008 29221915 PMC5963981

[B97] Wechsler D. (2011). *Wechsler Abbreviated Scale of Intelligence.* 2nd Edn. San Antonio, TX: NCS Pearson.

[B98] Weisleder A. Fernald A. (2013). Talking to children matters: early language experience strengthens processing and builds vocabulary. *Psychol. Sci.* 24 2143–2152. 10.1177/0956797613488145 24022649 PMC5510534

[B99] Weizman Z. O. Snow C. E. (2001). Lexical Input as related to children’s vocabulary acquisition: effects of sophisticated exposure and support for meaning. *Dev. Psychol.* 37 265–279. 10.1037/0012-1649.37.2.265 11269394

[B100] Wierenga L. M. Langen M. Oranje B. Durston S. (2014). Unique developmental trajectories of cortical thickness and surface area. *Neuroimage* 87 120–126. 10.1016/j.neuroimage.2013.11.010 24246495

[B101] Wilke M. Lidzba K. Krägeloh-Mann I. (2009). Combined functional and causal connectivity analyses of language networks in children: a feasibility study. *Brain Lang.* 108 22–29. 10.1016/j.bandl.2008.09.007 18952275

[B102] Yarkoni T. Braver T. S. (2010). “Cognitive neuroscience approaches to individual differences in working memory and executive control: conceptual and methodological issues,” in *Handbook of Individual Differences in Cognition* (New York, NY: Springer), 87–107.

[B103] Younger J. W. Lee K. W. Demir-Lira O. E. Booth J. R. (2019). Brain lateralization of phonological awareness varies by maternal education. *Dev. Sci.* 22:e12807. 10.1111/desc.12807 30735285

