# Supplementary material for: Parent Language Input Prior to School Forecasts Change in Children’s Language-Related Cortical Structures During Mid-Adolescence
Source: Front Hum Neurosci. 2021 Aug 2;15:650152. doi: 10.3389/fnhum.2021.650152 (PMC8366586; doi:10.3389/fnhum.2021.650152)
Supplement: Supplementary file 1 [file Table_1.DOCX]

**Supplementary materials**

**Bayesian approach**

To provide further support for our conclusions based on the frequentist approach, we also performed the model fitting process under a Bayesian paradigm using the rstanarm package in R. A number of factors led us to also include a Bayesian approach. The main reason was our small sample size. With smaller sample sizes, Bayesian methods are considered to provide less biased estimates and increased ability to detect non-null effects (McNeish & Stapleton, 2016). Thus, as an alternative to likelihood-based models, Bayesian Markov chain Monte Carlo (MCMC) estimation methods are becoming widespread. Unlike frequentist methods, Bayesian inference does not perform traditional hypothesis testing; rather, the focus is on maximizing the posterior distribution of the model parameters. In other words, Bayesian inference allows us to have an informed belief about our model parameters, based on the data that were observed. In Bayesian models, implementing sampling-based Bayesian methods such as MCMC, the inference is not limited by samples size but by the number of steps taken to approach infinity. This is of particular advantage in our present setting, given the small sample size (McNeish & Stapleton, 2016).

For our model, six MCMC sampling chains were used with 10000 iterations per chain, including a burn-in period (a burn-in period refers to the portion of the simulations used to inform where to sample next and typically defined as discarded iterations). The final stage of the model is to assign prior distributions for parameters. Uninformed priors were used to specify the model parameters. Diagnostics from the rstanarm package indicate that the effective sample sizes for each parameter were adequate, a metric necessary to ensure that the degree of autocorrelation between samples is low. Additionally, $R$^2^ values for all parameters were less than one, affirming the successful convergence of each chain. Lastly, none of the fit parameters had a Monte Carlo standard error greater than 10% of the posterior standard deviation. Parameter point estimates returned by the Bayesian model represent the posterior median of the parameter sampling distribution.

We used Bayesian credible intervals to test for significant differences: a 90% credible interval defines the region that the true effect of language input lies with 90% probability, given the data. We will consider a 90% credible interval that does not include zero to indicate a statistically significant association. However, for general transparency purposes, we will include 50 and 80% credible intervals in the tables as well. Figure S1 presents a visual representation for the plot of the posterior distribution – histogram of samples drawn from the posterior distribution for each parameter. The figure also provides a visual representation of the 90% and 50% credible intervals. To give an example of the interpretation of credible intervals, let’s take the effect of age. Table S1 represents the Bayesian equivalent of the model presented in Table 3 of the main manuscript. As seen in Table S1, the estimate for the median for age is 0.048, and also 90% credible interval for age is (0.016, 0.081). This is interpreted to suggest that under the Bayesian paradigm, having seen the observed data, there is over 90% probability that the true value of this parameter for age lies in this interval and thus the parameter is greater than 0. In other words, since 0 is not in the 90% credible interval, we can state that with reasonable post-data probability that cortical thickness increased over time. Overall, consistent with the frequentist approach, a nonlinear effect of age is observed.

Other parameters in the model are overall consistent with the frequentist linear mixed-model. We see that thickness in occipital regions was associated with greater cortical thickness in language areas, since none of the intervals include 0. Results suggested that controlling for other covariates, maternal IQ did not have an effect on cortical thickness since the majority of the sampling distribution includes 0. Using the 90% credible interval for SES, we can suggest that with reasonable post-data probability, SES was associated with greater thickness overall, and SES moderated the relations of age to cortical thickness, where higher SES children observed larger decreases in cortical thickness compared to lower SES children. With respect to sex, 90% credible intervals suggest that sex was a significant predictor of overall cortical thickness with females initially having greater cortical thickness but the effect decreasing over time. Similarly, 90% credible interval for scanner similarly suggests that scanner type did not predict cortical thickness.

Most importantly for our purposes, credible intervals suggest a high probability for language input to be a contributing factor to thickness. Specifically, language input moderated the relations of age to cortical thickness. We see that 50% credible interval for language input intercept, and 90% credible intervals for language input linear and quadratic term does not include 0. Under the Bayesian paradigm, then, we may claim that having seen the observed data, there is just over 90% probability that the true value of the parameters for linear and quadratic term relations do not include zero. In other words, the signs and effect sizes of the linear and quadratic terms for input suggest a concave upward parabola for individuals with a higher language input component. In other words, children with higher language input experienced larger decreases in cortical thickness in the 8-12 age range compared to children with lower language input. These results mirrors what we observed with the frequentist model.

**Table S1.**

*Results of a Bayesian analysis for the relationship between parental language input PCA (word tokens, decontextualized utterances) and cortical thickness. We controlled for the following covariates: child age, mean occipital thickness, sex, scanner type, mother IQ, family SES.*

|  | **Median** | **50% CI** | **80% CI** | **90% CI** |
| --- | --- | --- | --- | --- |
| Age | -0.03 | (-0.043, -0.018) | (-0.054, -0.006) | (-0.061, 0.001) |
| Age^2^ | 0.002 | (0, 0.004) | (-0.001, 0.005) | (-0.002, 0.006) |
| Mean occipital thickness | 0.088 | (0.082, 0.093) | (0.077, 0.098) | (0.074, 0.101) |
| Scanner | 0.018 | (-0.001, 0.037) | (-0.019, 0.055) | (-0.03, 0.065) |
| Mother IQ | -0.001 | (-0.003, 0) | (-0.004, 0.002) | (-0.005, 0.003) |
| SES composite | 0.024 | (0.004, 0.043) | (-0.014, 0.062) | (-0.025, 0.073) |
| SES composite X Age | -0.006 | (-0.008, -0.004) | (-0.009, -0.003) | (-0.01, -0.002) |
| Sex | 0.149 | (0.103, 0.194) | (0.062, 0.236) | (0.037, 0.261) |
| Sex Age | -0.071 | (-0.092, -0.05) | (-0.111, -0.032) | (-0.122, -0.02) |
| Sex Age^2^ | 0.007 | (0.004, 0.009) | (0.002, 0.011) | (0, 0.013) |
| Language input PCA | 0.06 | (0.035, 0.086) | (0.012, 0.109) | (-0.002, 0.123) |
| Language input PCA X Age | -0.034 | (-0.044, -0.024) | (-0.054, -0.015) | (-0.059, -0.009) |
| Language input PCA X Age^2^ | 0.004 | (0.003, 0.005) | (0.001, 0.006) | (0.001, 0.007) |

**Figure S1.**

*Figure presents a visual representation for the plot of the posterior distribution – histogram of samples drawn from the posterior distribution for each parameter. Each is bounded by two sets of dashed lines, with the blue and red lines representing the bounds of the 50% and 90% credible intervals, respectively. The vertical green line is present to indicate zero. Histogram A demonstrates the distribution of parameter values collected for the first principal component of language input. From the dashed lines, it appears that the value 0 lies just around the 50% credible interval constructed, while also falling within the bounds of the dashed line provided by the red. Histogram B demonstrates the distribution of parameter values for the language input and age and Histogram C represents the distribution parameter values for language input and quadratic age. For the latter, the value 0 lies outside the 90% credible interval constructed.*

|  | 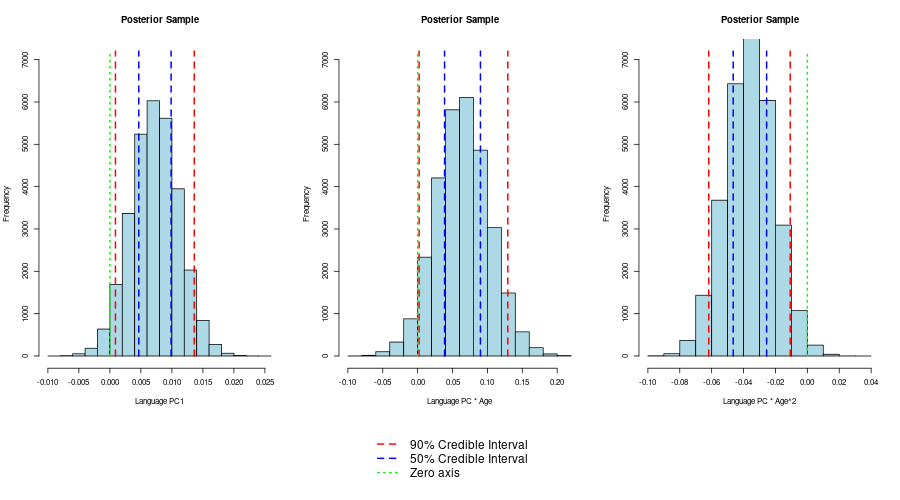 |  |
| --- | --- | --- |

**Region-specific relations using Bayesian approach**

Table S2 presents the exploratory Bayesian analysis including intercept, linear and quadratic term for each region of interest as fixed factors. The values for non-region relevant covariates, such as age, SES, and sex matched the previous main models. For the effect of language input, regions did differ from each other in that median credible intervals for some regions included 0 and the median estimates were close to 0, whereas other regions showed did not and this exhibited a stronger effect of language input. The regions where strongest relations to language input were observed were the following: IFG Opercularis, and STS – for these regions the 90% credible interval did not include 0. For IFG Opercularis and STS, similar to the main model, language input was positively related to thickness at baseline, negative to slope and positive to quadratic term. In other words, children with higher input had a higher intercept but observed steeper change over the time period observed.

## **Table S2**

*Results of a region-specific Bayesian analysis for the relationship between parental language input PCA (word tokens, decontextualized utterances) and cortical thickness. Each region of interest is included as a fixed factor. We controlled for the following covariates: child age, mean occipital thickness, sex, scanner type, mother IQ, family SES.*

|  | **Median** | **50% CI** | **80% CI** | **90% CI** |
| --- | --- | --- | --- | --- |
| Mother IQ | 0.088 | (0.083, 0.094) | (0.078, 0.099) | (0.075, 0.102) |
| Mean occipital thickness | 0.017 | (-0.002, 0.036) | (-0.019, 0.054) | (-0.029, 0.064) |
| Scanner | -0.028 | (-0.04, -0.015) | (-0.052, -0.004) | (-0.058, 0.003) |
| Age | 0.002 | (0, 0.003) | (-0.001, 0.005) | (-0.002, 0.006) |
| Age^2^ | 0.007 | (-0.013, 0.027) | (-0.032, 0.044) | (-0.045, 0.055) |
| SES composite | -0.006 | (-0.007, -0.004) | (-0.009, -0.002) | (-0.01, -0.001) |
| SES composite X Age | 0.164 | (0.118, 0.21) | (0.078, 0.253) | (0.053, 0.281) |
| Sex | -0.076 | (-0.097, -0.055) | (-0.115, -0.036) | (-0.126, -0.025) |
| Sex Age | 0.007 | (0.004, 0.01) | (0.002, 0.012) | (0.001, 0.013) |
| Sex Age^2^ | 3.056 | (2.928, 3.164) | (2.762, 3.256) | (2.582, 3.311) |
| IFG Opercularis | 0.101 | (0.067, 0.135) | (0.037, 0.165) | (0.019, 0.185) |
| IFG Opercularis X Language Input PCA | -0.052 | (-0.069, -0.036) | (-0.084, -0.021) | (-0.093, -0.013) |
| IFG Opercularis X Language Input PCA X Age | 0.006 | (0.004, 0.008) | (0.002, 0.01) | (0.001, 0.011) |
| IFG Opercularis X Language Input PCA X Age^2^ | 2.921 | (2.794, 3.03) | (2.628, 3.122) | (2.447, 3.177) |
| IFG Triangularis | 0.009 | (-0.025, 0.043) | (-0.055, 0.073) | (-0.072, 0.091) |
| IFG Triangularis X Language Input PCA | -0.008 | (-0.025, 0.008) | (-0.04, 0.023) | (-0.049, 0.031) |
| IFG Triangularis X Language Input PCA X Age | 0.001 | (-0.001, 0.003) | (-0.003, 0.005) | (-0.004, 0.006) |
| IFG Triangularis X Language Input PCA X Age^2^ | 3.011 | (2.884, 3.12) | (2.717, 3.211) | (2.536, 3.265) |
| SmG | 0.02 | (-0.013, 0.054) | (-0.043, 0.085) | (-0.062, 0.104) |
| SmG X Language Input PCA | -0.017 | (-0.033, -0.001) | (-0.048, 0.014) | (-0.057, 0.023) |
| SmG X Language Input PCA X Age | 0.002 | (0, 0.004) | (-0.002, 0.006) | (-0.003, 0.007) |
| SmG X Language Input PCA X Age^2^ | 3.229 | (3.1, 3.337) | (2.936, 3.428) | (2.756, 3.483) |
| STG | 0.082 | (0.048, 0.116) | (0.018, 0.146) | (0, 0.164) |
| STG X Language Input PCA | -0.05 | (-0.067, -0.034) | (-0.081, -0.019) | (-0.091, -0.011) |
| STG X Language Input PCA X Age | 0.005 | (0.003, 0.007) | (0.001, 0.009) | (0, 0.01) |
| STG X Language Input PCA X Age^2^ | 3.287 | (3.159, 3.395) | (2.993, 3.488) | (2.812, 3.542) |
| MTG | 0.067 | (0.034, 0.101) | (0.002, 0.132) | (-0.016, 0.15) |
| MTG X Language Input PCA | -0.031 | (-0.048, -0.015) | (-0.063, 0) | (-0.072, 0.009) |
| MTG X Language Input PCA X Age | 0.004 | (0.002, 0.006) | (0, 0.007) | (-0.001, 0.008) |
| MTG X Language Input PCA X Age^2^ | 2.694 | (2.566, 2.802) | (2.401, 2.893) | (2.22, 2.949) |
| STS | 0.122 | (0.088, 0.155) | (0.057, 0.186) | (0.04, 0.205) |
| STS X Language Input PCA | -0.053 | (-0.07, -0.037) | (-0.085, -0.022) | (-0.093, -0.013) |
| STS X Language Input PCA X Age | 0.005 | (0.003, 0.007) | (0.002, 0.009) | (0, 0.01) |
| STS X Language Input PCA X Age^2^ | -0.074 | (-0.107, -0.042) | (-0.137, -0.011) | (-0.156, 0.008) |

**Table S3.**

*First-order correlations between parental word tokens and decontextualized utterances at different time points.*

|  |  | **Word Tokens** | | | **Decontextualized Utterances** | | |
| --- | --- | --- | --- | --- | --- | --- | --- |
|  |  | **18m** | **30m** | **42m** | **18m** | **30m** | **40m** |
| **Word Tokens** | 18m | 1.00 | 0.68 | 0.53 | 0.84 | 0.42 | 0.12 |
|  | 30m |  | 1.00 | 0.61 | 0.57 | 0.70 | 0.38 |
|  | 42m |  |  | 1.00 | 0.51 | 0.71 | 0.73 |
| **Decontextualized utterances** | 18m |  |  |  | 1.00 | 0.45 | 0.19 |
|  | 30m |  |  |  |  | 1.00 | 0.75 |
|  | 42m |  |  |  |  |  | 1.00 |

* *p* < .05.

**Table S4.**

*Results of a linear mixed model analysis for the relationship between parental language input PCA (word tokens, decontextualized utterances, rare words) and cortical thickness. We controlled for the following covariates: child age, mean occipital thickness, sex, scanner type, mother IQ, family SES.*

|  | **Estimate** | **Std. Error** | **95%L** | **95%U** | **p-val** |
| --- | --- | --- | --- | --- | --- |
| Intercept | 3.1958 | 0.1583 | 2.8855 | 3.5061 | < .001** |
| Age | -0.0358 | 0.0178 | -0.0708 | -0.0009 | 0.0449* |
| Age^2^ | 0.0024 | 0.0022 | -0.0019 | 0.0068 | 0.2761 |
| Mean occipital thickness | 0.0871 | 0.008 | 0.0715 | 0.1027 | < .001** |
| Scanner | 0.018 | 0.0283 | -0.0376 | 0.0735 | 0.526 |
| Mother IQ | -0.0012 | 0.0021 | -0.0054 | 0.003 | 0.5964 |
| SES composite | 0.0258 | 0.027 | -0.0271 | 0.0787 | 0.3497 |
| SES composite X Age | -0.0063 | 0.0026 | -0.0114 | -0.0012 | 0.0152* |
| Sex | 0.1205 | 0.061 | 0.001 | 0.2401 | 0.0524 |
| Sex Age | -0.0586 | 0.0285 | -0.1145 | -0.0026 | 0.0407* |
| Sex Age^2^ | 0.0054 | 0.0035 | -0.0015 | 0.0123 | 0.1244 |
| Language input PCA | 0.0408 | 0.0324 | -0.0226 | 0.1043 | 0.211 |
| Language input PCA X Age | -0.0259 | 0.0132 | -0.0518 | 0 | 0.0503 |
| Language input PCA X Age^2^ | 0.003 | 0.0016 | -0.0002 | 0.0062 | 0.0643 |

**Table S5.**

*Results of a linear mixed model analysis for the relationship between parental language input PCA (word tokens and decontextualized utterances) and cortical thickness in 17 children who have at least 2 scans. We controlled for the following covariates: child age, mean occipital thickness, sex, scanner type, mother IQ, family SES. Inferential statistics include estimate, standard error, 95% CI upper and lower limit and p-value.*

|  | **Estimate** | **Std. Error** | **95%L** | **95%U** | **p-val** |
| --- | --- | --- | --- | --- | --- |
| Intercept | 3.1697 | 0.1656 | 2.8452 | 3.4943 | < 0.001* |
| Age | -0.02 | 0.0222 | -0.0635 | 0.0236 | 0.369 |
| Age^2^ | 0.0017 | 0.0028 | -0.0038 | 0.0071 | 0.5531 |
| Mean occipital thickness | 0.0874 | 0.0082 | 0.0714 | 0.1035 | < 0.001* |
| Scanner | 0.0006 | 0.0291 | -0.0565 | 0.0576 | 0.9849 |
| Mother IQ | -0.0006 | 0.0023 | -0.0052 | 0.0039 | 0.7914 |
| SES composite | 0.0384 | 0.0312 | -0.0228 | 0.0995 | 0.2372 |
| SES composite X Age | -0.0061 | 0.003 | -0.012 | -0.0002 | 0.042* |
| Sex | 0.0978 | 0.0841 | -0.0671 | 0.2627 | 0.2539 |
| Sex Age | -0.0785 | 0.0359 | -0.1489 | -0.008 | 0.0293* |
| Sex Age^2^ | 0.007 | 0.0043 | -0.0015 | 0.0155 | 0.1063 |
| Language input PCA | 0.0233 | 0.0426 | -0.0602 | 0.1069 | 0.5887 |
| Language input PCA X Age | -0.037 | 0.0159 | -0.0682 | -0.0059 | 0.02* |
| Language input PCA X Age^2^ | 0.0039 | 0.0019 | 0.0002 | 0.0076 | 0.0382* |

**Table S6.**

*Results of a linear mixed model analysis for the relationship between parental language input PCA (word tokens and decontextualized utterances) and cortical thickness. We controlled for PPVT as well as the following covariates: child age, mean occipital thickness, sex, scanner type, mother IQ, family SES. Inferential statistics include estimate, standard error, 95% CI upper and lower limit and p-value.*

|  | **Estimate** | **Std. Error** | **95%L** | **95%U** | **p-val** |
| --- | --- | --- | --- | --- | --- |
| Intercept | 3.1914 | 0.1656 | 2.8668 | 3.516 | <0.001* |
| Age | -0.024 | 0.0193 | -0.0618 | 0.0139 | 0.2149 |
| Age^2^ | 0.0014 | 0.0024 | -0.0033 | 0.0061 | 0.5493 |
| Mean occipital thickness | 0.0883 | 0.0081 | 0.0726 | 0.1041 | <0.001* |
| Scanner | -0.0194 | 0.0157 | -0.0501 | 0.0113 | 0.2164 |
| Mother IQ | 0.014 | 0.0285 | -0.0418 | 0.0699 | 0.622 |
| PPVT | -0.0014 | 0.0023 | -0.0059 | 0.0031 | 0.5515 |
| SES composite | 0.0223 | 0.029 | -0.0345 | 0.0791 | 0.4502 |
| SES composite X Age | -0.0038 | 0.0031 | -0.0098 | 0.0022 | 0.2177 |
| Sex | 0.1627 | 0.0678 | 0.0297 | 0.2956 | 0.0194* |
| Sex Age | -0.0824 | 0.0323 | -0.1458 | -0.019 | 0.0111* |
| Sex Age^2^ | 0.0076 | 0.0039 | -0.0001 | 0.0153 | 0.0544 |
| Language input PCA | 0.0669 | 0.0379 | -0.0075 | 0.1412 | 0.0816 |
| Language input PCA X Age | -0.0374 | 0.0157 | -0.0681 | -0.0066 | 0.0176* |
| Language input PCA X Age^2^ | 0.004 | 0.0019 | 0.0003 | 0.0077 | 0.0332* |

**Table S7.**

*Results of a region-specific linear mixed-model analysis for the relationship between parental language input PCA (word tokens, decontextualized utterances) and cortical thickness. Each region of interest is included as a fixed factor. We controlled for the following covariates: child age, mean occipital thickness, sex, scanner type, mother IQ, family SES.*

|  | **Estimate** | **Std. Error** | **95%L** | **95%U** | **p-val** |
| --- | --- | --- | --- | --- | --- |
| Mother IQ | -0.0011 | 0.0022 | -0.0054 | 0.0032 | 0.62 |
| Mean occipital thickness | 0.0873 | 0.008 | 0.0716 | 0.103 | <0.001* |
| Scanner | 0.0178 | 0.0283 | -0.0378 | 0.0733 | 0.5306 |
| Age | -0.0289 | 0.0189 | -0.0659 | 0.0081 | 0.1261 |
| Age^2^ | 0.0018 | 0.0024 | -0.0029 | 0.0065 | 0.452 |
| SES composite | 0.0218 | 0.0283 | -0.0337 | 0.0772 | 0.4501 |
| SES composite X Age | -0.0056 | 0.0027 | -0.0109 | -0.0003 | 0.0369* |
| Sex | 0.1533 | 0.0666 | 0.0227 | 0.2839 | 0.0245* |
| Sex Age | -0.074 | 0.0316 | -0.1359 | -0.0122 | 0.0194* |
| Sex Age^2^ | 0.0069 | 0.0039 | -0.0007 | 0.0145 | 0.0762 |
| IFG Opercularis | 3.2009 | 0.1368 | 2.9327 | 3.469 | <0.001* |
| IFG Opercularis X Language Input PCA | 0.0977 | 0.0496 | 0.0005 | 0.1949 | 0.0501 |
| IFG Opercularis X Language Input PCA X Age | -0.0528 | 0.0246 | -0.1011 | -0.0045 | 0.0325* |
| IFG Opercularis X Language Input PCA X Age^2^ | 0.0062 | 0.003 | 0.0004 | 0.0121 | 0.0375* |
| IFG Triangularis | 3.0665 | 0.1368 | 2.7984 | 3.3347 | <0.001* |
| IFG Triangularis X Language Input PCA | 0.005 | 0.0496 | -0.0922 | 0.1022 | 0.9203 |
| IFG Triangularis X Language Input PCA X Age | -0.0082 | 0.0246 | -0.0565 | 0.0401 | 0.7387 |
| IFG Triangularis X Language Input PCA X Age^2^ | 0.0012 | 0.003 | -0.0046 | 0.007 | 0.688 |
| SmG | 3.1562 | 0.1368 | 2.8881 | 3.4244 | <0.001* |
| SmG X Language Input PCA | 0.0168 | 0.0496 | -0.0804 | 0.114 | 0.7347 |
| SmG X Language Input PCA X Age | -0.0171 | 0.0246 | -0.0654 | 0.0312 | 0.4879 |
| SmG X Language Input PCA X Age^2^ | 0.0019 | 0.003 | -0.004 | 0.0077 | 0.5305 |
| STG | 3.3735 | 0.1368 | 3.1053 | 3.6416 | <0.001* |
| STG X Language Input PCA | 0.078 | 0.0496 | -0.0192 | 0.1751 | 0.1173 |
| STG X Language Input PCA X Age | -0.0503 | 0.0246 | -0.0986 | -0.002 | 0.0417* |
| STG X Language Input PCA X Age^2^ | 0.0051 | 0.003 | -0.0008 | 0.0109 | 0.0892 |
| MTG | 3.4322 | 0.1368 | 3.1641 | 3.7004 | <0.001* |
| MTG X Language Input PCA | 0.0631 | 0.0496 | -0.0341 | 0.1603 | 0.2044 |
| MTG X Language Input PCA X Age | -0.0311 | 0.0246 | -0.0794 | 0.0172 | 0.2071 |
| MTG X Language Input PCA X Age^2^ | 0.0036 | 0.003 | -0.0022 | 0.0094 | 0.2279 |
| STS | 2.8393 | 0.1368 | 2.5711 | 3.1074 | <0.001* |
| STS X Language Input PCA | 0.1185 | 0.0496 | 0.0213 | 0.2157 | 0.0177* |
| STS X Language Input PCA X Age | -0.0536 | 0.0246 | -0.1019 | -0.0053 | 0.03* |
| STS X Language Input PCA X Age^2^ | 0.0055 | 0.003 | -0.0004 | 0.0113 | 0.0681 |

**Figure S2**

*Individual trajectories and mean observed thickness by group on the left and model fit for each region of interest on the right. In both figures the solid line represents the Loess curve fit on the observation. High and low language PCA groups are separated by median language input PCA value.*

***
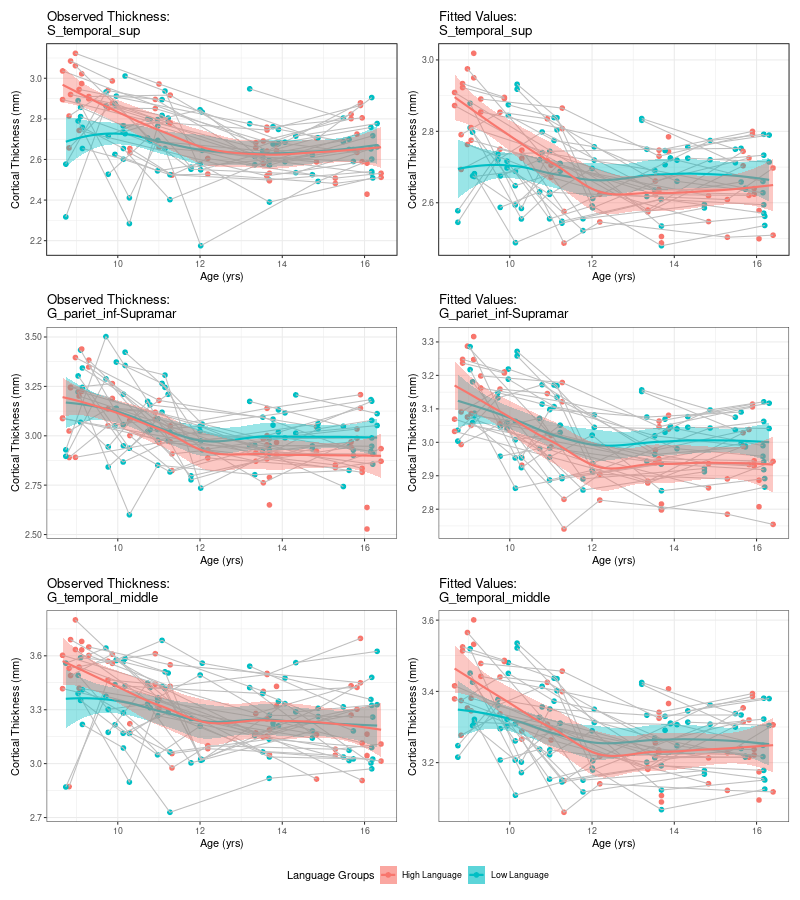
***

**
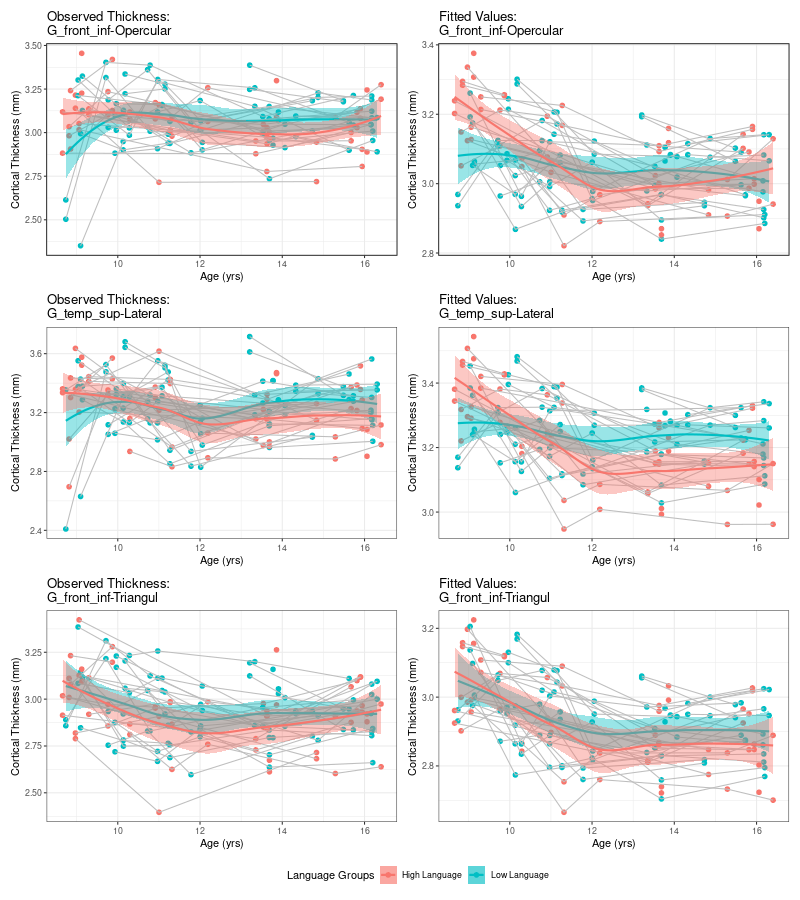
**

**References**

McNeish, D. M., & Stapleton, L. M. (2016). The effect of small sample size on two-level model estimates: A review and illustration. *Educational Psychology Review, 28*(2), 295-314.
